# Supplementary material for: Tubular Endogenous Erythropoietin Protects Renal Function against Ischemic Reperfusion Injury
Source: Int J Mol Sci. 2024 Jan 19;25(2):1223. doi: 10.3390/ijms25021223 (PMC10816907; doi:10.3390/ijms25021223)
Supplement: Supplementary file 1 [file ijms-25-01223-s001.zip › ijms-2764590-supplementary.pdf]

Supplementary file

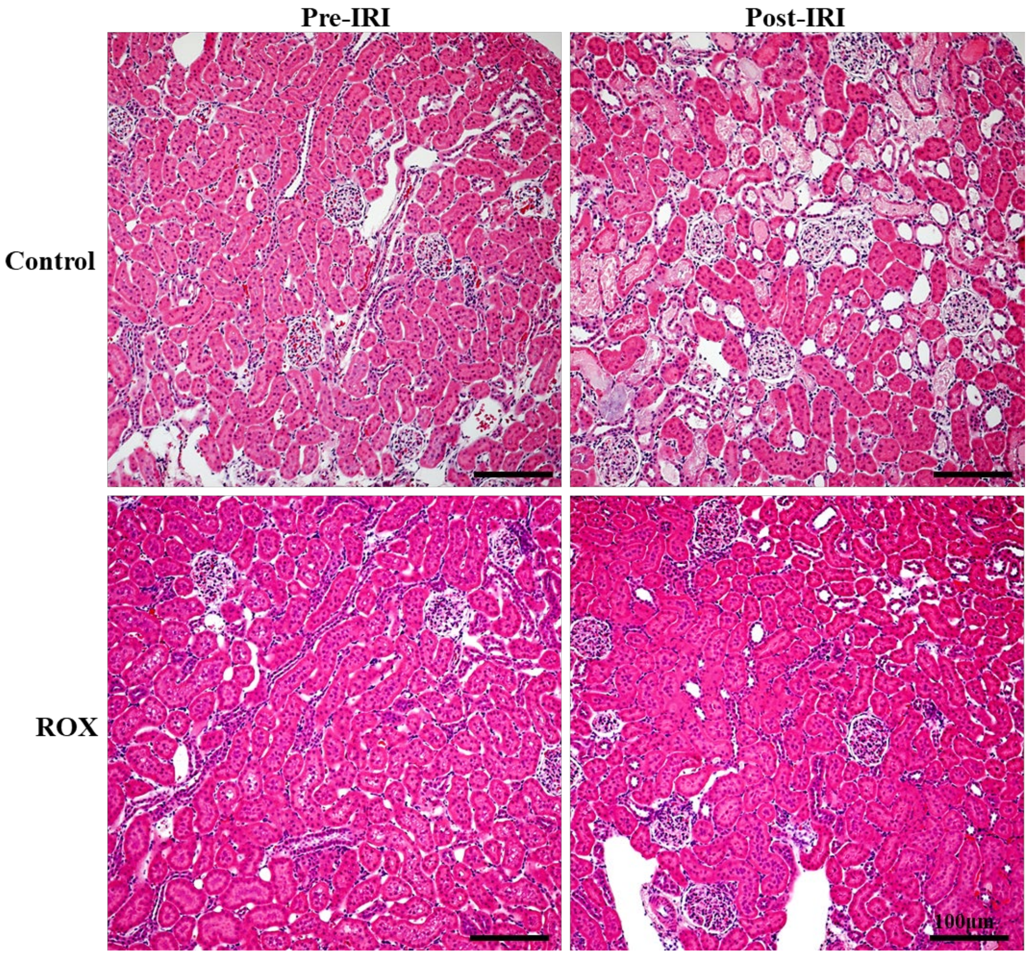

Supplementary Figure S1. HE staining of kidney pre- and post-IRI. Proximal tubules were damaged by IRI. Glomerulus was not damaged by IRI. 24 hr ROX decreased the damages in proximal tubules.
